# Effects of mesenchymal stem cell-derived nanovesicles in experimental allergic airway inflammation

**DOI:** 10.1186/s12931-023-02310-y

**Published:** 2023-01-05

**Authors:** Elga Bandeira, Su Chul Jang, Cecilia Lässer, Kristina Johansson, Madeleine Rådinger, Kyong-Su Park

**Affiliations:** Krefting Research Centre, Department of Internal Medicine and Clinical Nutrition, Institute of Medicine, Sahlgrenska Academy, University of Gothenburg, Gothenburg, Sweden

**Keywords:** Extracellular vesicles, Nanovesicles, Mesenchymal stem cells, Asthma, Anti-inflammation

## Abstract

**Background:**

Allergic asthma is associated with airflow obstruction and hyper-responsiveness that arises from airway inflammation and remodeling. Cell therapy with mesenchymal stem cells (MSC) has been shown to attenuate inflammation in asthma models, and similar effects have recently been observed using extracellular vesicles (EV) obtained from these cells. Biologically functional vesicles can also be artificially generated from MSC by extruding cells through membranes to produce EV-mimetic nanovesicles (NV). In this study, we aimed to determine the effects of different MSC-derived vesicles in a murine model of allergic airway inflammation.

**Methods:**

EV were obtained through sequential centrifugation of serum-free media conditioned by human bone marrow MSC for 24 h. NV were produced through serial extrusion of the whole cells through filters. Both types of vesicles underwent density gradient purification and were quantified through nanoparticle tracking analysis. C57BL/6 mice were sensitized to ovalbumin (OVA, 8 µg), and then randomly divided into the OVA group (intranasally exposed to 100 µg OVA for 5 days) and control group (exposed to PBS). The mice were then further divided into groups that received 2 × 10^9^ EV or NV (intranasally or intraperitoneally) or PBS immediately following the first OVA exposure.

**Results:**

Administration of EV and NV reduced cellularity and eosinophilia in bronchoalveolar lavage (BAL) fluid in OVA-sensitized and OVA-exposed mice. In addition, NV treatment resulted in decreased numbers of inflammatory cells within the lung tissue, and this was associated with lower levels of Eotaxin-2 in both BAL fluid and lung tissue. Furthermore, both intranasal and systemic administration of NV were effective in reducing inflammatory cells; however, systemic delivery resulted in a greater reduction of eosinophilia in the lung tissue.

**Conclusions:**

Taken together, our results indicate that MSC-derived NV significantly reduce OVA-induced allergic airway inflammation to a level comparable to EV. Thus, cell-derived NV may be a novel EV-mimetic therapeutic candidate for treating allergic diseases such as asthma.

**Supplementary Information:**

The online version contains supplementary material available at 10.1186/s12931-023-02310-y.

## Background

Asthma is a chronic inflammatory disease associated with airflow obstruction, bronchial hyper-responsiveness, and respiratory symptoms such as wheezing and shortness of breath [1]. Despite the broad and heavy impact of asthma worldwide, there are relatively few treatment options for patients with poorly controlled asthma despite being treated with inhaled corticosteroids and long-acting β-agonists [2]. Importantly, inhaled corticosteroids do not have any intrinsic capacity to induce the repair of structural changes caused by the remodeling of the asthmatic airways. Therefore, therapeutic approaches to both attenuate inflammation and improve remodeling processes may have a place in clinical management.

In the search for a therapy that can both reduce inflammation and improve remodeling of the airways, mesenchymal stem cells (MSC) have shown promise as a new strategy for attenuating inflammation and restoring balance to the repair processes thus preventing noxious remodeling [3]. MSC are capable of promoting immunomodulatory effects in a wide range of respiratory illnesses, even in xenogeneic transplantation models [4]. Nevertheless, treatment with whole cells comes with some concerns because whole cells may begin to proliferate and differentiate in the recipient patient.

It has previously been shown that conditioned medium from MSC possesses similar therapeutic effects as whole MSC, partly due to the presence of extracellular vesicles (EV) [5, 6]. These vesicles are ubiquitously secreted by cells in culture, even without stimulation, and they contain specifically sorted molecules including subcellular structures, proteins, and nucleic acids. Much interest has been focused on these vesicles due to their ability to promote changes in target cells and tissues [7]. Alternatively, EV-mimetic nanovesicles (NV) can be generated through serial extrusions of cells [8]. The contents of these vesicles are a random assortment of molecules, but they can still trigger responses in target cells similarly to EV. Importantly, the biological membrane contents of NV suggest that they might be a suitable alternative to EV with the advantage of higher yields [8]. Vesicle-based therapies may overcome the safety concerns related to treatment using whole cells and may reduce the need for repeated invasive procedures.

Previous studies have shown that EV derived from human MSC have therapeutic properties in a model of allergic airway inflammation [5, 9], but no report exists describing whether NV derived from these cells have similar anti-inflammatory effects as EV. This study therefore aimed to compare the efficacy of manufactured NV to that of EV isolated from human MSC on inflammation in an experimental model of allergic airway inflammation induced by ovalbumin (OVA) and to further explore whether local or systemic administration of these vesicles has any therapeutic advantages.

## Methods

### Cell culture and vesicle isolation protocols

Human primary bone marrow-derived MSC were obtained from Texas A&M Health Science Center College of Medicine Institute for Regenerative Medicine [10]. The cells were cultivated in alpha MEM medium supplemented with 15% fetal bovine serum (HyClone Laboratories) and 1% antibiotic solution (100 units/ml penicillin and 100 mg/ml streptomycin, HyClone Laboratories), and all cells were used between the third and fifth passages. We followed a commonly used protocol for the isolation of EV through ultracentrifugation of the conditioned media followed by density gradient purification of the samples [11]. For NV isolation, after 24 h of incubation in serum-free medium the cells were washed with phosphate-buffered saline (PBS, pH 7.4) and detached from the cell culture flasks using PBS containing 10 mM EDTA. MSC were resuspended at a density of 5 × 10^6^ cells per mL in a total of 10 mL of PBS. The cell suspensions were passed five times through a series of polycarbonate membrane filters (Whatman) with pore sizes of 10 µm, 5 µm and, 1 µm using a mini-extruder (Avanti Polar Lipids). A total of 1 mL 50% iodixanol (Axis-Shield PoC AS) and 2 mL of 10% iodixanol was sequentially added to each 10 mL ultracentrifuge tube followed by 7 mL of the cell suspension effluent from the membrane filter. The layers formed between 50% iodixanol and 10% iodixanol after ultracentrifugation at 100,000 × *g* for 2 h were collected and considered NV.

### Characterization of EV and NV

EV and NV were dispersed in PBS at the optimal concentration for measuring, and then particle concentrations were measured using ZetaView analyzer (Particle Metrix GmbH, software version 8.2.30.1). Measurements were assessed in duplicate, and each individual data set was obtained from two stationary layers with 11 measurements in each layer. Camera sensitivity was set at 80% in all measurements. For transmission electron microscopy (TEM), formvar/carbon-coated copper grids (Ted Pella, Inc., Redding, CA, USA) were glow discharged before the samples were loaded. The grids and samples were incubated for 15 min before being fixed in 2% paraformaldehyde and in 2.5% glutaraldehyde with PBS washes in between. The samples were then washed in dH2O and then contrasted in 2% uranyl acetate. The preparations were examined using a LEO 912AB Omega electron microscope (Carl Zeiss NTS, Jena, Germany).

### Gene and protein analysis of EV and NV

RNA from EV or NV was isolated using a miRCURY RNA Isolation Kit for Biofluids (Exiqon) according to the manufacturer’s protocol. DNA was isolated using a Qiamp DNA Blood Mini kit (Qiagen) according to the manufacturer’s protocol. One microliter of isolated RNA and DNA was analyzed for its quality, yield, and nucleotide length by capillary electrophoresis using an Agilent RNA 6000 Picochip and Agilent High sensitivity DNA chip, respectively, on an Agilent 2100 Bioanalyzer (Agilent Technologies). The protein content was measured using a Qubit Protein Assay Kit in a Qubit fluorometer (ThermoFisher Scientific) according to the manufacturer’s instructions.

### Western blot analysis

EV or NV (10 µg) was separated by 10% SDS-PAGE and shifted to a polyvinylidene difluoride membrane. The membrane blocked in nonfat milk was incubated with anti-flotillin-1 antibody (Santa Cruz Biotechnology), anti-CD81 antibody (Santa Cruz Biotechnology), or anti-beta-actin antibody (Santa Cruz Biotechnology). The specific bands were detected with chemiluminescent substrates following treatment with secondary antibody conjugated with horseradish peroxidase.

### Animal model and experimental protocol

**Fig. 1:**
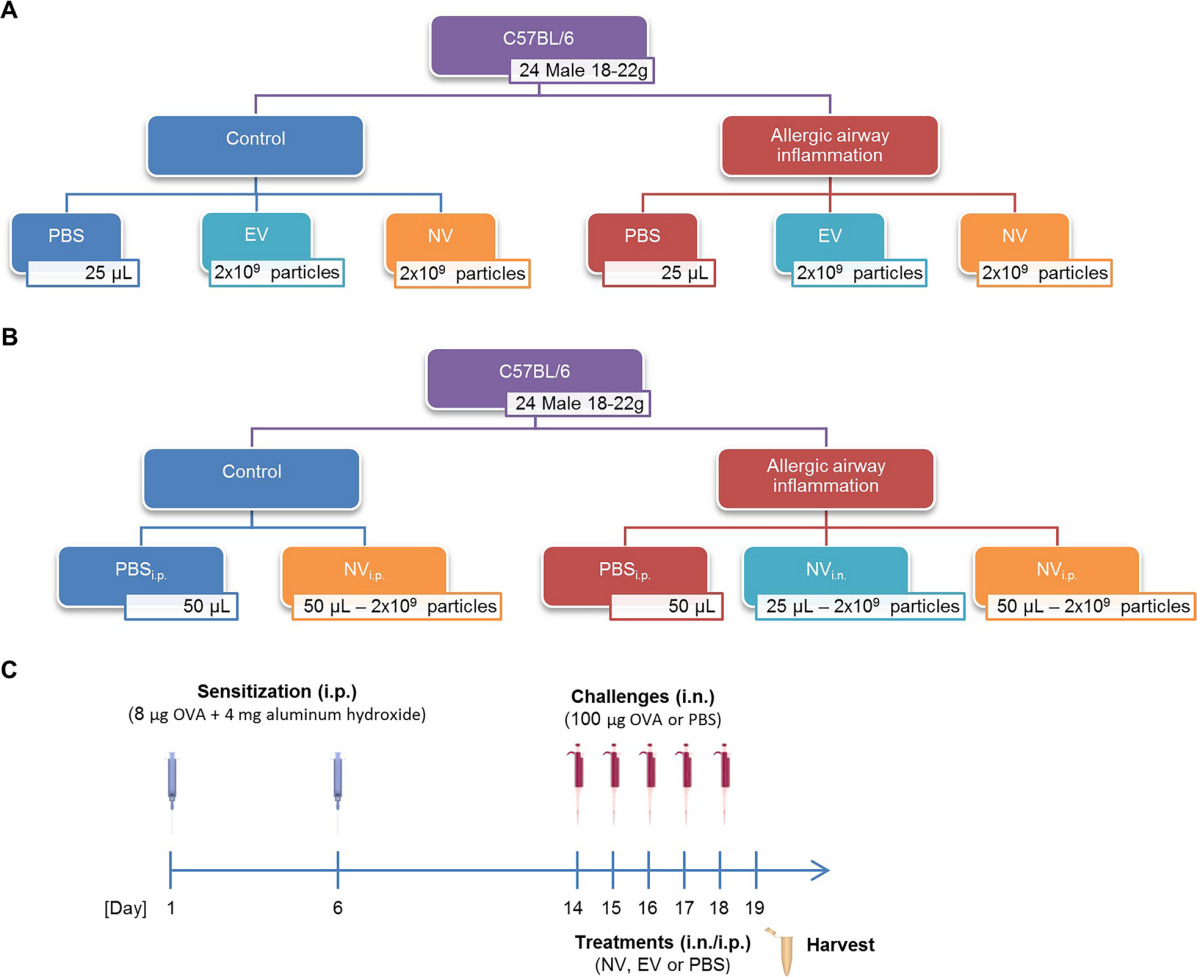
Experimental design for the investigation of the therapeutic activity of EV and NV. **A** Distribution of the experimental groups to compare EV and NV. **B** Distribution of experimental groups to compare NV administration routes. **C** Timeline of the asthma experimental model

Sixty C57BL/6 male mice (8–12 weeks old) were purchased from Charles River and were housed at Experimental Biomedicine at the University of Gothenburg, Sweden. The study was approved by the local Animal Ethics Committee (permit no. 89-2016, 126/14 and 22/16) and conducted in adherence with institutional animal use and care guidelines. The experimental model of allergic inflammation was created following the protocol described in previous work from our group [12]. Briefly, the mice were sensitized with two intraperitoneal (i.p.) injections of a solution containing 8 µg of OVA (Sigma) and 4 mg of aluminum hydroxide in 250 µL of PBS. The animals were then randomly divided into groups that received intranasal (i.n.) challenges with 25 µL of plain PBS (control groups) or 100 µg of OVA in 25 µL of PBS (OVA groups). Thirty minutes after the first challenge, the two groups were both further divided into groups that either received 25 µL of PBS and/or received 2 × 10^9^ NV or the same amount of EV via i.n. administration (Fig. 1A). Alternatively, the animals were given NV (in 50 µL of PBS) or PBS via i.p. administration (Fig. 1B). The animals were OVA challenged once a day for 5 days and then euthanized 24 h after the last OVA challenge for the collection of bronchoalveolar lavage (BAL) fluid and lung tissues for further analysis (Fig. 1C).

### Inflammation assessment

Sample collection was performed as previously described in detail in Boberg et al*.* [13]. Briefly, BAL fluid was collected from tracheotomized mice by instillation of sterile PBS followed by gentle aspiration. Mediators were analyzed in cell-free BAL, and BAL cells were processed for differential cell counting. Specifically, the levels of Eotaxin-2 in the cell-free BAL were determined using a commercial ELISA kit (R&D Systems). Cytospins were made from the BAL cells, and these were stained with Giemsa and analyzed under a light microscope at 200 × magnification.

For single-cell suspensions from lung tissues, a fraction of the lung was collected and weighed and the tissue was mechanically disrupted using a Miltenyi GentleMacs Dissociator and digested with 1 mg/mL of collagenase D (Roche) and 40 U/mL of DNase I. The cells were collected and incubated with antibodies against the following surface markers: CD3—conjugated with FITC, B220—conjugated with PE, CD45—conjugated with APC, CD4—conjugated with APC-H7, and CD25—conjugated with BV421 (all purchased from BD Biosciences). Flow cytometry was performed using a FACSVerse Flow Cytometer (BD Biosciences) running BD FACSuite Software and analyzed with FlowJo Software (TreeStar Inc.).

### IL-10 release by macrophages

RAW 264.7 cells were seeded in a 24-well plate, and then EV or NV (1 × 10^9^) was treated for 24 h to examine cytokine production in the cell supernatants. And then, IL-10 was measured by DuoSet ELISA Development kit (R&D Systems).

### Biodistribution analysis

For whole body biodistribution evaluation, NV were incubated with Cy7 monoNHS ester (5 µM, Amersham Biosciences) for 2 h at 37 °C. Cy7-labeled NV were isolated using iodixanol density gradient ultracentrifugation (10% and 50% iodixanol) at 100,000 × *g* for 2 h. Cy7-labeled NV (8 × 10^9^) were given via i.p. or i.n. administration to C57BL/6 mice. After 30 min, 3 h, 6 h, and 24 h, Cy7 fluorescence throughout the whole body of the mice was visualized with an IVIS system (Caliper Life Sciences). In addition, fluorescence signals in the liver, heart, kidney, spleen, lung, pancreas, and intestines were detected. For confocal microscopy, NV were stained with DiI (1 mM DiI in DMSO/PBS, Life Technologies) and then given via i.n. administration. Lungs were embedded in optimum cutting temperature (OCT) compound followed by immediate snap-freezing and storage at − 80 °C until sectioning using a Leica CM1900 UV cryostat (Leica Microsystems Nussloch GmbH, Solms, Germany).

### Cellular uptake of NV

EV or NV were stained with DiO (Molecular Probes) for 30 min at 37 ˚C. Macrophage cell line (RAW 264.7 and MH-S) was labeled with Cellmask Deep Red (Thermo Fisher Scientific) followed by treated with DiO-labeled EV or NV for 6 h. The macrophage cell was fixed with 4% paraformaldehyde, permeabilized with 0.2% Triton X-100, and observed by a fluorescence light microscope (Zeiss Axio observer; Carl Zeiss). Also, flow cytometry was analyzed using BD FACSVerse Flow Cytometer running BD FACSuit Software (BD Biosciences) and FlowJo Software (Tree Star Inc.).

### Statistical analysis

All results are expressed as the mean and standard error of the mean (SEM). One-way ANOVA followed by Tukey’s multiple comparison test was used to assess the differences between groups, and *P* < 0.05 was considered to be significant). If the values did not pass the normality test with Shapiro–Wilk normality test, we performed Kruskal–Wallis with multiple comparison test. All of the statistical analyses were performed using GraphPad Prism 7.0.1 (GraphPad Software Inc.).

## Results

### EV and biomimetic NV characterization

**Fig. 2:**
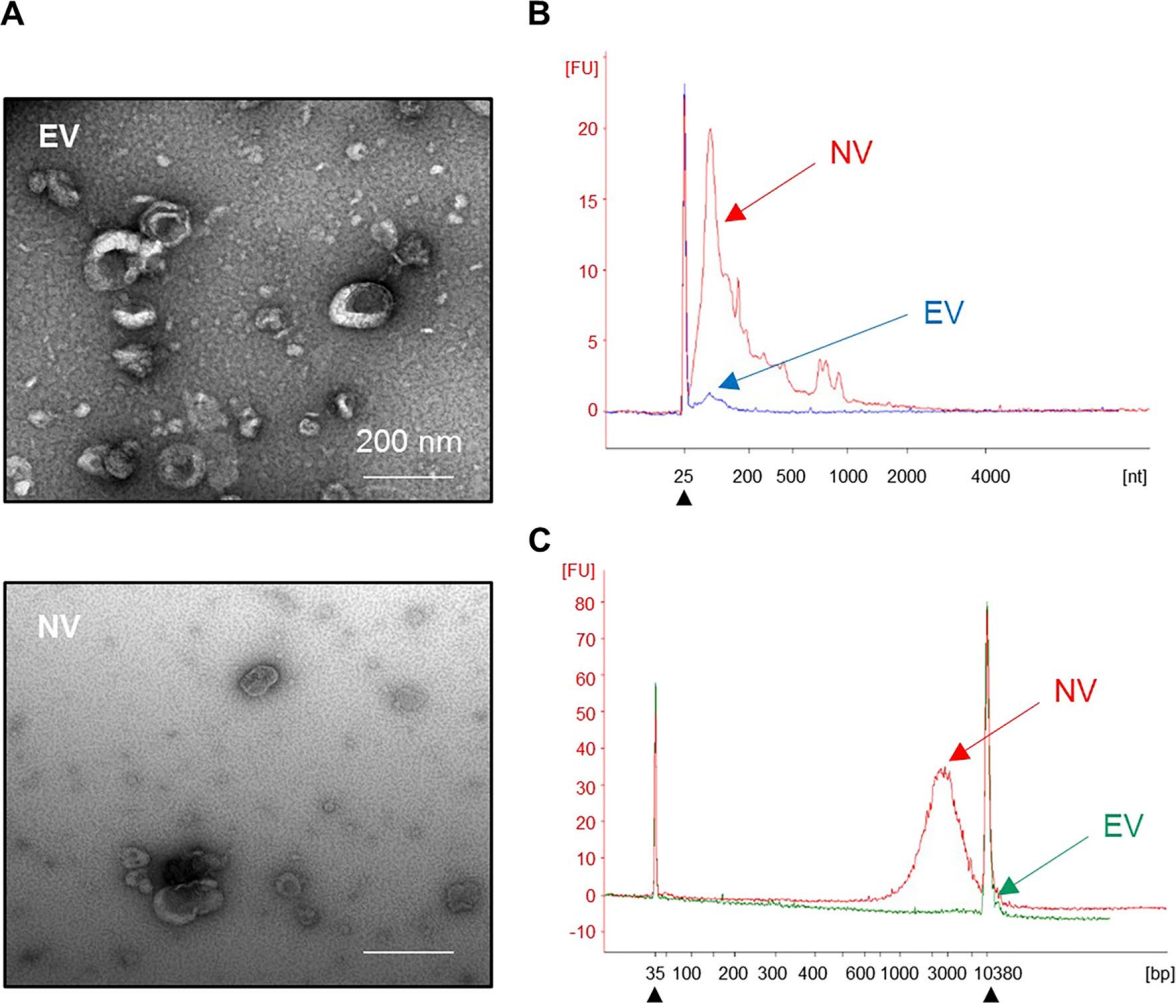
Characterization of MSC-derived NV versus EV. **A** TEM images of EV and NV. Scale bars, 200 nm. **B** RNA content of EV (blue) and NV (red). **C** DNA content of EV (green) and NV (red). Filled triangles indicate internal markers

When comparing EV and NV morphology, both vesicles presented with similar shapes and sizes as seen by TEM (Fig. 2A). Measurement of both particles with a nanoparticle tracking analyzer showed a mode that varied from 120 to 140 nm for both types of vesicles (Data not shown). Both types of vesicles contained small RNA (< 200 nucleotides; Fig. 2B). While NV presented with DNA, EV did not exhibit detectable DNA (Fig. 2C). Moreover, NV presented with higher particle per protein ratios than EV (Data not shown). Moreover, we showed that NV present similar expression pattern as EV when it comes to markers such as Flotillin-1, CD81, and beta-actin (Additional file 1: Fig. S1), which are markers typically used to characterize MSC-EV [14, 15].

### Comparing the immunomodulatory effects of EV and NV

**Fig. 3:**
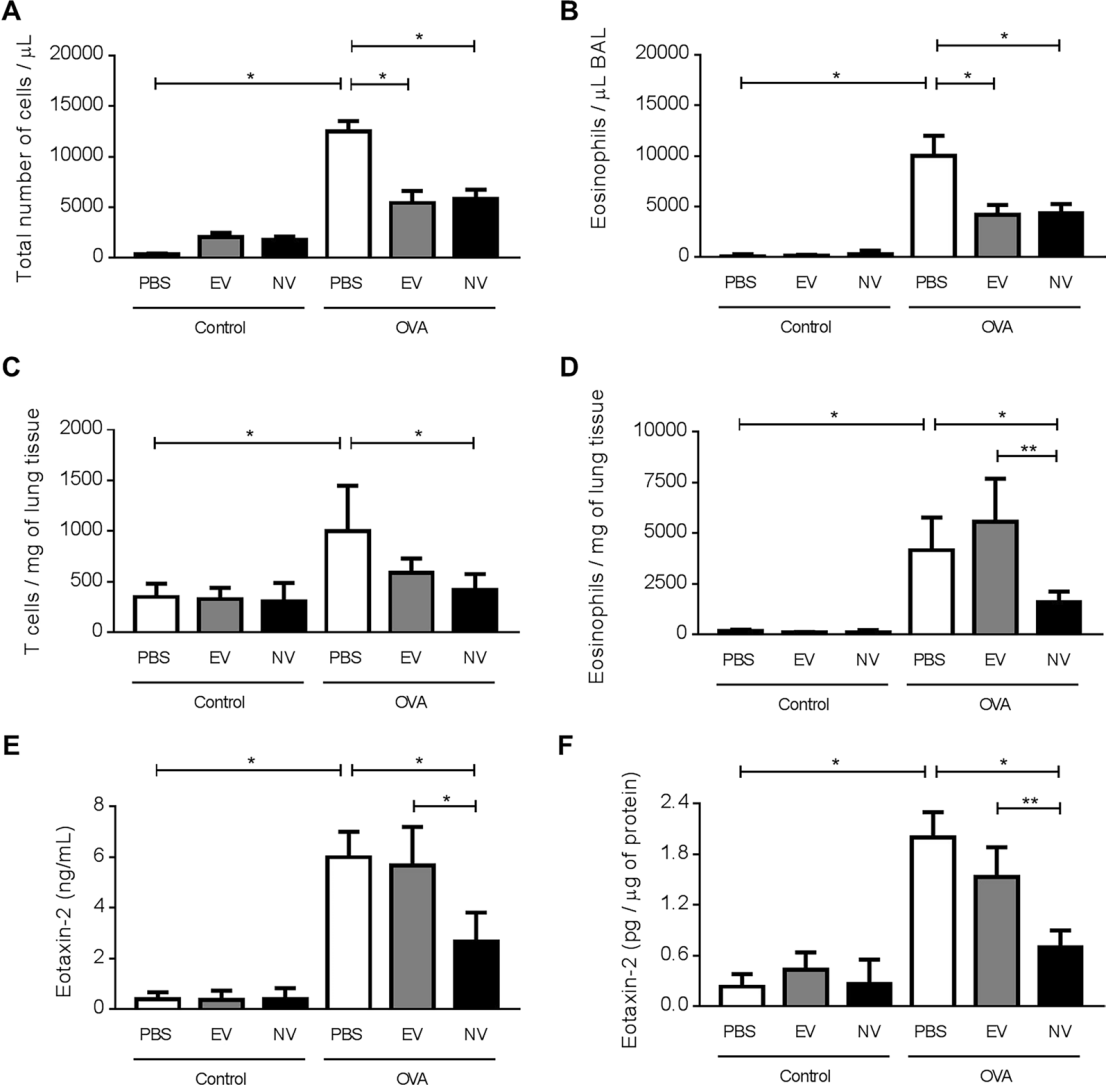
Treatment with MSC-derived NV resulted in decreased airway inflammation in BAL fluid and lung tissue compared to EV treatment. **A**, **B** The total number of inflammatory cells (**A**) and eosinophils (**B**) in BAL fluid. **C**, **D** The number of T cells (**C**) and eosinophils (**D**) that infiltrated into the lung tissues. **E**, **F** The level of Eotaxin-2 in BAL fluid (**E**) and lung tissues (**F**). Data are presented as the mean ± SEM. *P < 0.05, **P < 0.01 by one-way ANOVA with Tukey’s post test (n = 5)

To determine whether MSC-derived NV can reduce allergic airway inflammation in a mouse model, OVA and aluminum hydroxide were given via i.p. injection for sensitization, and then OVA and vesicles were given as an i.n. challenge as described in Fig. 1. No significant alterations in cellular and molecular parameters were seen after administration of EV or NV in PBS-exposed control animals (Fig. 3). Mice in the OVA group that received multiple doses of EV or NV presented with reduced numbers of inflammatory cells in the BAL fluid, particularly eosinophils, when compared to those treated with PBS (Fig. 3A, B). Nevertheless, NV were significantly more efficient than EV at reducing the number of total T lymphocytes (Fig. 3C), especially Th2 cells (Additional file 1: Fig. S2), and eosinophils (Fig. 3D) within the lung tissue. This result is supported by the finding of reduced lung levels of Eotaxin-2, which is a factor that has chemotactic effects on eosinophils [16], after treatment with NV but not after treatment with EV (Fig. 3E, F). Levels of IL-5 and Eotaxin-1 were not affected by either treatment (Data not shown). Photomicrographs of the lung parenchyma showed that the model successfully accomplished inflammation of the airways, but none of the treatment strategies tested in this study were able to revert this inflammation, possibly promoting a heterogeneous response across the lung (Additional file 1: Fig. S3).

### Comparing different strategies for NV therapy

**Fig. 4:**
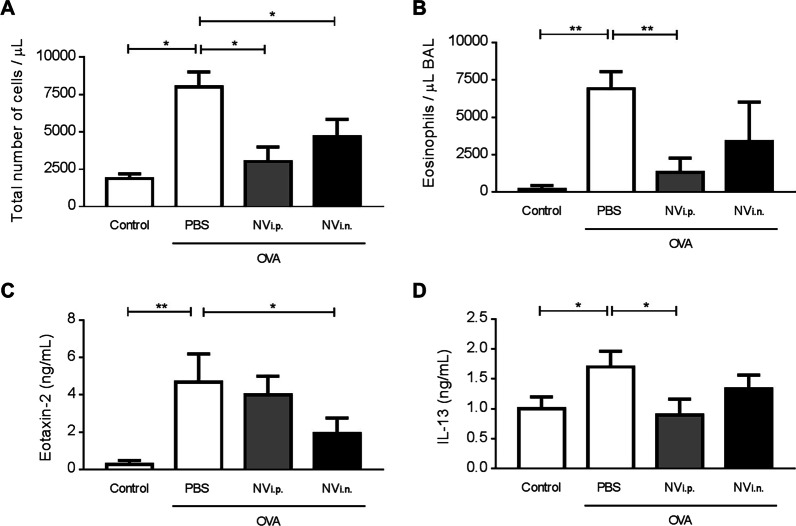
Comparison of different administration strategies for NV therapy. **A**, **B** OVA-sensitized/challenged mice were given NV by i.p. or i.n. administration, and the total numbers of inflammatory cells (**A**) and eosinophils (**B**) were measured in BAL fluid. **C**, **D** The levels of Eotaxin-2 (**C**) and IL-13 **(D**) in BAL fluid. Data are presented as the mean ± SEM. *P < 0.05, **P < 0.01 by one-way ANOVA with Tukey’s post test (n = 5)

Because we observed that NV had a similar or better effect than EV, we wanted to further compare the efficacy of NV treatment when administered through two different routes, namely local (i.n.) versus systemic (i.p.) administration. Both routes were effective at reducing the total number of inflammatory cells in BAL fluid (Fig. 4A); however, i.p. delivery resulted in a more significant reduction of eosinophil infiltration in the BAL fluid (Fig. 4B).

In contrast with the i.n. instillation of NV, the i.p. treatment yielded a reduction in IL-13, but not Eotaxin-2, in the BAL fluid of the treated animals (Fig. 4C, D). Both of systemic and local treatment with NV reduced the expression of IFN-γ in lung tissue lysate to similar levels as controls, whereas there was no effect on the levels of IL-4 (Additional file 1: Fig. S4).

### Comparing the biodistribution of NV according to different administration routes

**Fig. 5:**
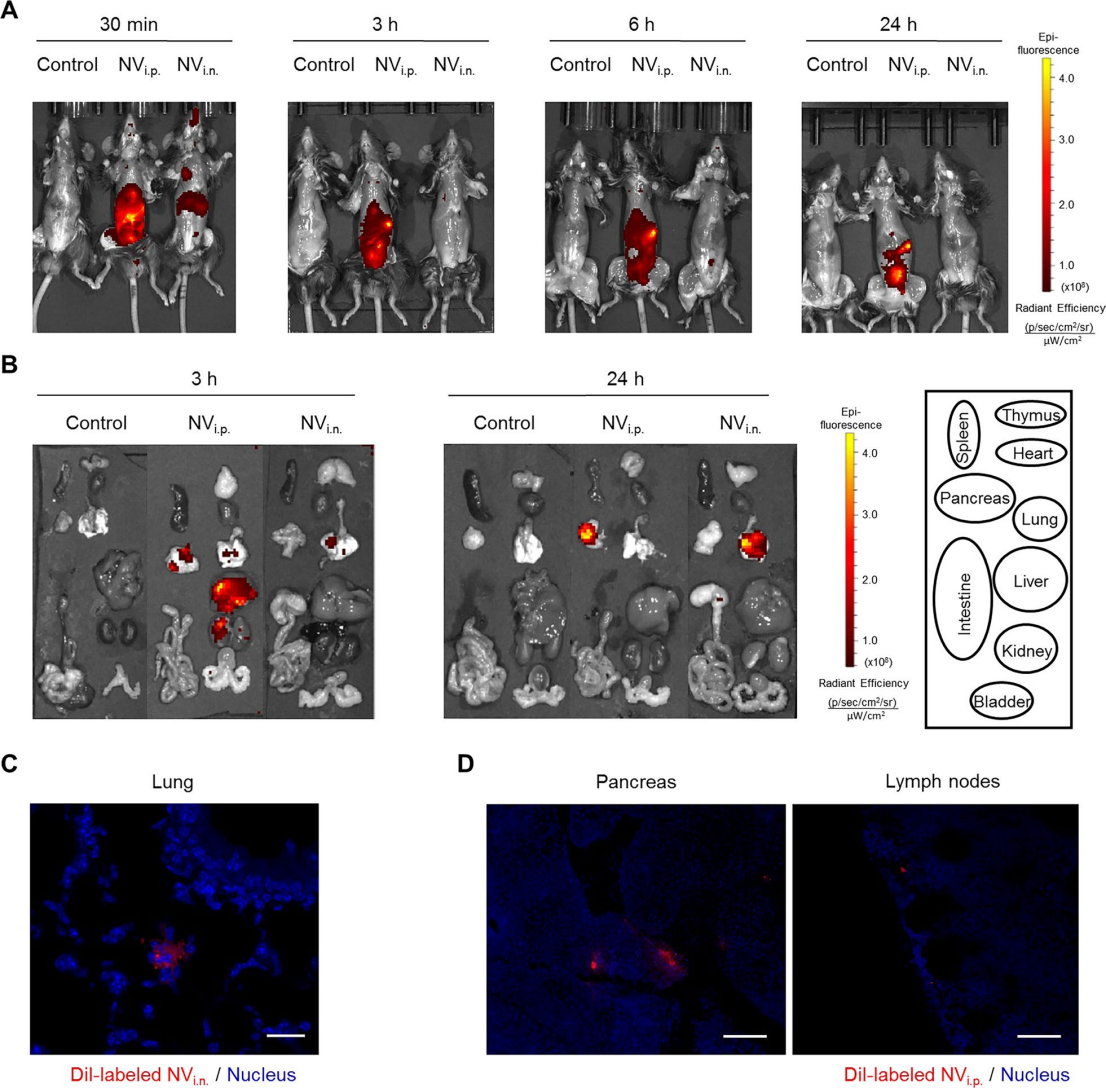
Biodistribution analysis of NV in mice using near-infrared imaging. **A** NV labeled with Cy7 were tracked through infrared in vivo imaging at 30 min, 3 h, 6 h, and 24 h after i.p. or i.n. administration in mice. **B** Various tissues were collected at 3 h and 24 h following administration of Cy7-labeled NV. **C** Internalization of NV (red) in lung tissue after i.n. administration. **D** Internalization of NV (red) in the pancreas and lymph nodes after i.p. administration. Scale bars, 100 µm

To further assess the possible distinct targets of NV therapy through the two different administration routes, we analyzed the biodistribution of Cy7-stained NV on the whole body and organ level (Fig. 5A, B). Our analysis indicated that when administered intranasally, the labeled NV were distributed in the lung parenchyma and were retained there for at least 24 h. Small amounts of labelled NV were also detected in the stomachs of those animals, suggesting partial swallowing of the NV dose given by the i.n. route. In contrast, i.p. administration of NV did not result in any detectable accumulation of dye in the lung parenchyma or airways at 24 h. Instead, the dye was found in the liver, kidney and pancreas after 3 h, but most of the fluorescence had disappeared by 24 h except for pancreas, suggesting elimination through the urine.

At the histological level, we observed the internalization of NV stained with DiI through confocal microscopy in accordance with the organ distribution. At 3 h after administration, NV could be detected in the lung tissue of animals that received i.n. NV (Fig. 5C) and in the pancreas and lymph nodes of animals that received i.p. NV (Fig. 5D). As macrophages are the predominant immune effector cells resident in the alveolar space [17], we next compared the uptake profile of EV or NV in this macrophage cells. As a result, the uptake efficiency of EV and NV was comparable each other in both RAW 264.7 cells (Additional file 1: Fig. S5A, B) and MH-S cells (Additional file 1: Fig. S5C), suggesting that EV and NV are dealt with similarly by the recipient cells.

Given that IL-10 reduces Th2 cytokine secretion as well as eosinophilia in allergic mice [18], we further explored whether EV or NV could produce this anti-inflammatory cytokine on macrophages. NV induced significantly IL-10 production from RAW 264.7 cells similarly with EV (Additional file 1: Fig. S6), implying that secondary mediators from EV or NV-treated macrophages could partly contribute to dampen allergic inflammation, although most vesicles disappear from the tissue.

## Discussion

In this study, we show that NV derived from MSC exhibit similar morphological and molecular characteristics as EV and that they have therapeutic immunomodulatory activities in a mouse model of airway inflammation induced by OVA sensitization and challenge. Specifically, we demonstrate that the NV can reduce excessive airway inflammation in mice through a significant decrease in eosinophil infiltration and cytokine production in BAL fluid and lung tissues. In addition, i.n. administration of NV shows a different anti-inflammatory pattern compared to systemic administration. Moreover, in mice treated with NV i.n., the vesicles were accumulated especially in the lung, which is consistent with the anti-inflammatory profile. Collectively, these data support the hypothesis that MSC-derived NV can contribute to significantly diminishing OVA-induced lung inflammation in vivo by blocking specific inflammatory mediators.

We found that when NV were produced from bone marrow-derived MSC, they exhibited similar shapes and diameters as naturally produced EV, which has also been shown in previous studies [19, 20]. Importantly, our group has previously reported that MSC-derived NV harbor a specific subset of proteins that are also common proteins in natural EV [8]. For example, NV also have EV marker proteins such as Flotillin-1 and CD81 as well as other top-100 EV markers as determined by Western blot and proteomic analysis. This suggests that NV and EV at least partly originate from similar compartments of MSC membranes. We also confirm that both NV and EV significantly reduce airway eosinophilic inflammation induced by OVA (Fig. 3). Determining which vesicular molecules contribute to the vesicle-mediated protection remains a challenging but important task.

There are several reports showing that natural EV have anti-inflammatory biological activity in the lung. For example, Zhu et al*.* reported that MSC-derived EV were therapeutically bioactive in endotoxin-induced lung injury, and this beneficial effect seemed to be partially mediated by growth factors present in the EV [21]. Also, MSC-derived EV were shown to attenuate allergic airway remodeling via immunomodulation on pulmonary macrophage [22, 23]. Moreover, specific EV cargo such as miR-146a-5p has been reported to be involved in preventing allergic airway inflammation [24]. However, to date no study has specifically determined the therapeutic activity of extruded NV from MSC on lung diseases, specifically in any allergic asthma model. Because there are still hurdles for the clinical use of EV due to their relatively low yield and costly isolation process [25], it is of great importance to determine whether NV have similar therapeutic effects as EV. We confirm here that NV can be generated with a higher yield than natural EV and that they have therapeutic potential that is comparable to EV. Taken together, these findings suggest that MSC-derived NV are scalable, efficient, and potentially cost-effective anti-inflammatory vesicles derived from MSC.

Modern management of asthma is focused on reducing airway inflammation through the long-term use of inhaled corticosteroids [26]. However, although inhaled corticosteroids are recognized as highly effective therapeutics for asthma control, they are not curative. Kerzerho et al*.* compared the therapeutic effect of systemic versus inhaled corticosteroid treatment in allergic asthma and confirmed that systemic administration is slightly more effective in preventing the development of airway inflammation [27]. In the same context, we assumed in the present study that the route of administration of NV affects the therapeutic activity of NV on the reduction of lung inflammation. Administration of NV is likely to be effective independently of the administration route, but the systemic exposure yielded more pronounced immunomodulation (Fig. 4). This might be due to different cell targeting according to the different routes of administration. The i.p. route seems to affect primarily the immune system and potentially lymphocytes based on the observed reduction in IL-13, while the i.n. route is more directed towards airway epithelial cells and the reduction in Eotaxin-2. These observations may help to guide the decision of route of administration in different types of inflammatory lung diseases.

Our group has previously reported that MSC-derived NV can distribute throughout the whole body when given via i.p. administration, but they seem to ultimately accumulate in various organs such as the lung, liver, and kidney [8]. Consistent with this observation, the currently reported near infrared imaging of NV fluorescence indicated that i.p. administration of NV also distribute throughout whole body but accumulate a little more in the pancreas and lymph nodes, which are closely related to the role of adaptive immunity in asthma progression [28]. However, when mice were given i.n. administration of fluorescent NV, the NV signals were mainly localized in the lung, which supports our data showing the significant inhibition of Eotaxin-2 by i.n. administration of NV. Naturally occurring EV are generally known to be internalized into recipient cells through multiple mechanisms such as endocytosis, micropinocytosis, and phagocytosis [29], and based on our biodistribution analysis NV can be expected to move across epithelial or endothelial barriers to target various cells or tissues. However, the exact uptake mechanism of NV into epithelial and immune cells has not yet been elucidated, and this should be further studied to improve our understanding of the therapeutic mechanism of action of NV.

Several mechanisms have been described for the therapeutic properties of MSC treatment in asthma. For example, MSC have been shown to alter the profile of macrophages by inducing the presentation of the anti-inflammatory phenotype (M2) instead of the pro-inflammatory phenotype (M1) [30]. Also, it was found that IL-10 is a crucial mediator secreted by macrophages to reduce inflammation through the inhibition of eosinophilia [31], and MSC-derived NV have also been shown to reduce bacteria-induced pro-inflammatory cytokines through upregulation of IL-10 [8]. In addition to the direct therapeutic effects of NV, immune cell-derived mediators induced by NV might play a significant anti-inflammatory role in the airway. Thus, further studies should clarify which cytokines including IL-10 are primarily involved in NV-mediated protection.

## Conclusions

Our study demonstrates for the first time the immunomodulatory activity of NV in an OVA-sensitized airway inflammatory animal model and suggests different therapeutic activities depending on the route of NV administration. Moreover, our overall data suggest that EV-mimetic NV from MSC potentially have a small advantage for clinical translation due to their high yields. However, there is still safety concerns with high doses of NV, or long-term treatment, because NV contains some DNA fragments. For future NV development, it is possible to eliminate contaminating protein and nucleotides by enzymatic or osmotic treatment [32]. Moreover, further development of NV derived from MSC from various tissue sources should be considered in order to improve the clinical efficacy of current treatments for many human diseases, including asthma.

## Supplementary Information

**Additional file 1: Figure S1.** A Identification of vesicular markers on EV or NV (10 µg) by Western blot analysis**. B** Full-length of blot images of the Western blots shown in Fig. S1A. **Figure S2.** The relative percentage of Th2 cells in the lungs of EV or NV-treated mice. Data are presented as the mean ± SEM. *P < 0.05, **P < 0.01 by one-way ANOVA with Tukey’s post test (n = 5). **Figure S3.** Effects of EV and NV on airway inflammation in the asthma model. A, B Hematoxylin and eosin-stained lung sections (A) and inflammation scores (B) in animals challenged with OVA and subsequently treated with EV or NV. Magnifications: 400 × . Data are presented as the mean ± SEM. **Figure S4.** The different cytokine profiles in the lungs of NV-treated mice depending on the administration route. A, B The levels of IL-4 (A) and IFN-γ (B) in the lung tissues. Data are presented as the mean ± SEM. *P < 0.05, **P < 0.01 by one-way ANOVA with Tukey’s post test (n = 5). **Figure S5.** The uptake profile of EV or NV in the macrophage cell line. A DiO-labeled EV or NV (green) was treated to RAW 264.7 cells for 6 h. Cell membranes (red) and nucleus (blue) were stained with Cellmask Deep Red and DAPI. Scale bars, 10 µm. B, C The uptake efficiency of both vesicles by RAW 264.7 cells (B) and MH-S cells (C) was analyzed by flow cytometry. The results are indicated by the relative percentage of DiO-positive cells. Data are presented as the mean ± SEM. ***P < 0.001; ns, not significant, by one-way ANOVA with Tukey’s post test (n = 5). **Figure S6.** IL-10 production by EV or NV in the macrophage cell line. RAW 264.7 cells were incubated with EV or NV (10^9^) for 24 h, and then the concentration of IL-10 in the conditioned medium was measured. Data are presented as the mean ± SEM. ***P < 0.001 by one-way ANOVA with Tukey’s post test (n = 3).

## Data Availability

The datasets used and/or analyzed during the current study are available from the corresponding author on reasonable request.

## Abbreviations

MSC: Mesenchymal stromal cell
EV: Extracellular vesicle
NV: Nanovesicle
OVA: Ovalbumin
PBS: Phosphate-buffered saline
BAL: Bronchoalveolar lavage
TEM: Transmission electron microscopy
i.n.: Intranasal
i.p.: Intraperitoneal
