# Supplementary material for: Effects of mesenchymal stem cell-derived nanovesicles in experimental allergic airway inflammation
Source: Respir Res. 2023 Jan 5;24:3. doi: 10.1186/s12931-023-02310-y (PMC9817274; doi:10.1186/s12931-023-02310-y)
Supplement: Supplementary file 1 — Additional file 1: Figure S1. A Identification of vesicular markers on EV or NV (10 µg) by Western blot analysis. B Full-length of blot images of the Western blots shown in Fig. S1A. Figure S2. The relative percentage of Th2 cells in the lungs of EV or NV-treated mice. Data are presented as the mean ± SEM. *P < 0.05, **P < 0.01 by one-way ANOVA with Tukey’s post test (n = 5). Figure S3. Effects of EV and NV on airway inflammation in the asthma model. A, B Hematoxylin and eosin-stained lung sections (A) and inflammation scores (B) in animals challenged with OVA and subsequently treated with EV or NV. Magnifications: 400 × . Data are presented as the mean ± SEM. Figure S4. The different cytokine profiles in the lungs of NV-treated mice depending on the administration route. A, B The levels of IL-4 (A) and IFN-γ (B) in the lung tissues. Data are presented as the mean ± SEM. *P < 0.05, **P < 0.01 by one-way ANOVA with Tukey’s post test (n = 5). Figure S5. The uptake profile of EV or NV in the macrophage cell line. A DiO-labeled EV or NV (green) was treated to RAW 264.7 cells for 6 h. Cell membranes (red) and nucleus (blue) were stained with Cellmask Deep Red and DAPI. Scale bars, 10 µm. B, C The uptake efficiency of both vesicles by RAW 264.7 cells (B) and MH-S cells (C) was analyzed by flow cytometry. The results are indicated by the relative percentage of DiO-positive cells. Data are presented as the mean ± SEM. ***P < 0.001; ns, not significant, by one-way ANOVA with Tukey’s post test (n = 5). Figure S6. IL-10 production by EV or NV in the macrophage cell line. RAW 264.7 cells were incubated with EV or NV (109) for 24 h, and then the concentration of IL-10 in the conditioned medium was measured. Data are presented as the mean ± SEM. ***P < 0.001 by one-way ANOVA with Tukey’s post test (n = 3). [file 12931_2023_2310_MOESM1_ESM.docx]

**
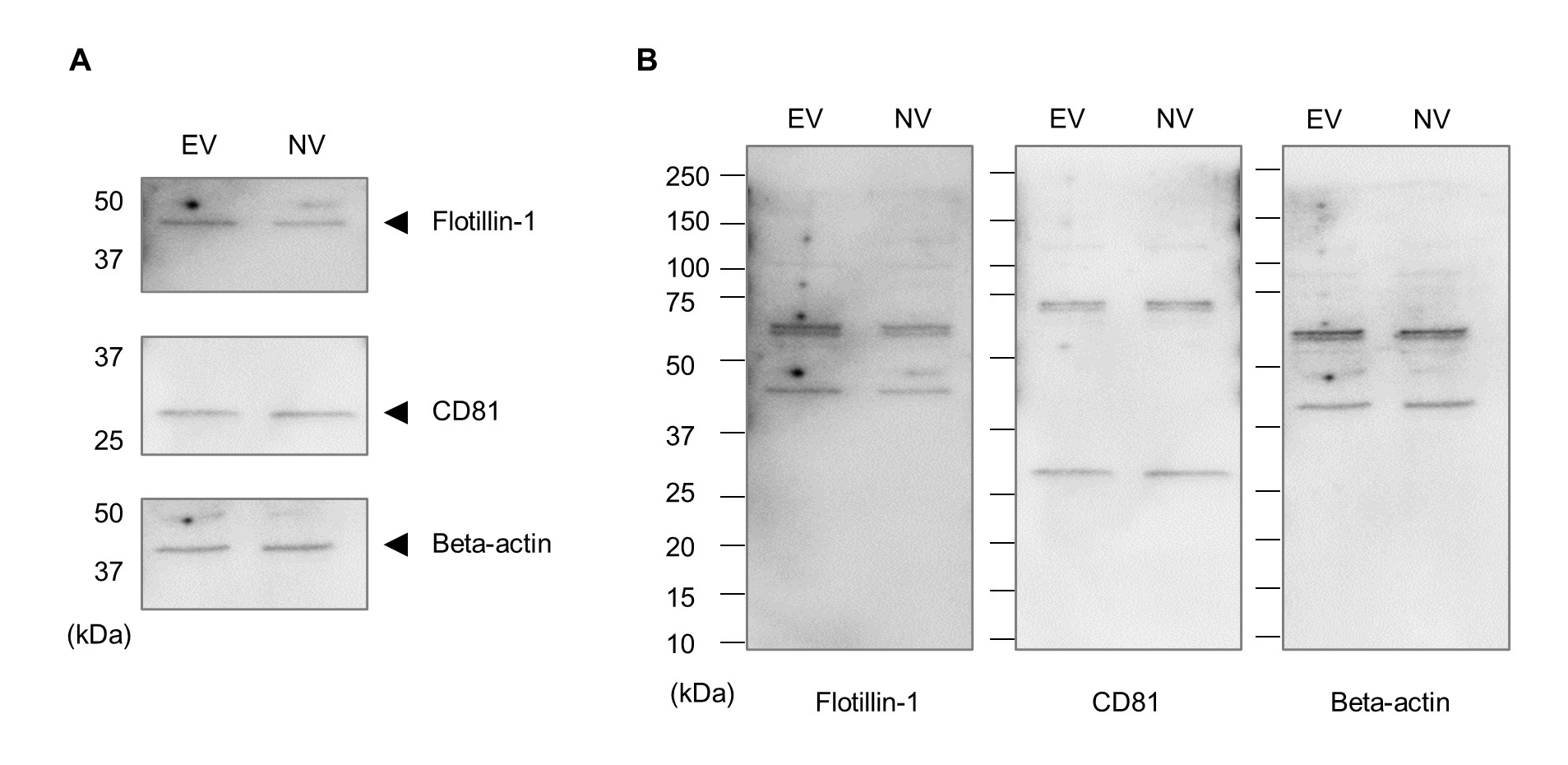
**

**Figure S1 A** Identification of vesicular markers on EV or NV (10 µg) by Western blot analysis. **B** Full-length of blot images of the Western blots shown in Fig. S1A.

**
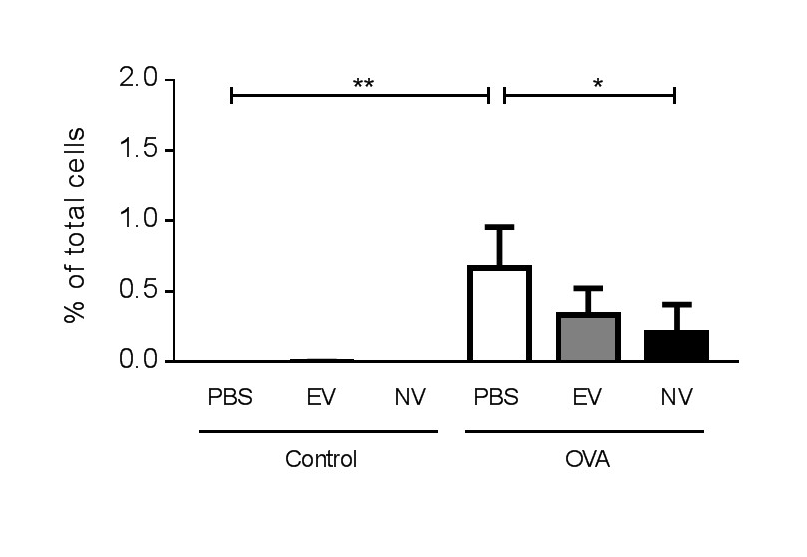
**

**Figure S2** The relative percentage of Th2 cells in the lungs of EV or NV-treated mice. Data are presented as the mean ± SEM. **P* < 0.05, ***P* < 0.01 by one-way ANOVA with Tukey’s post test (n = 5).

**
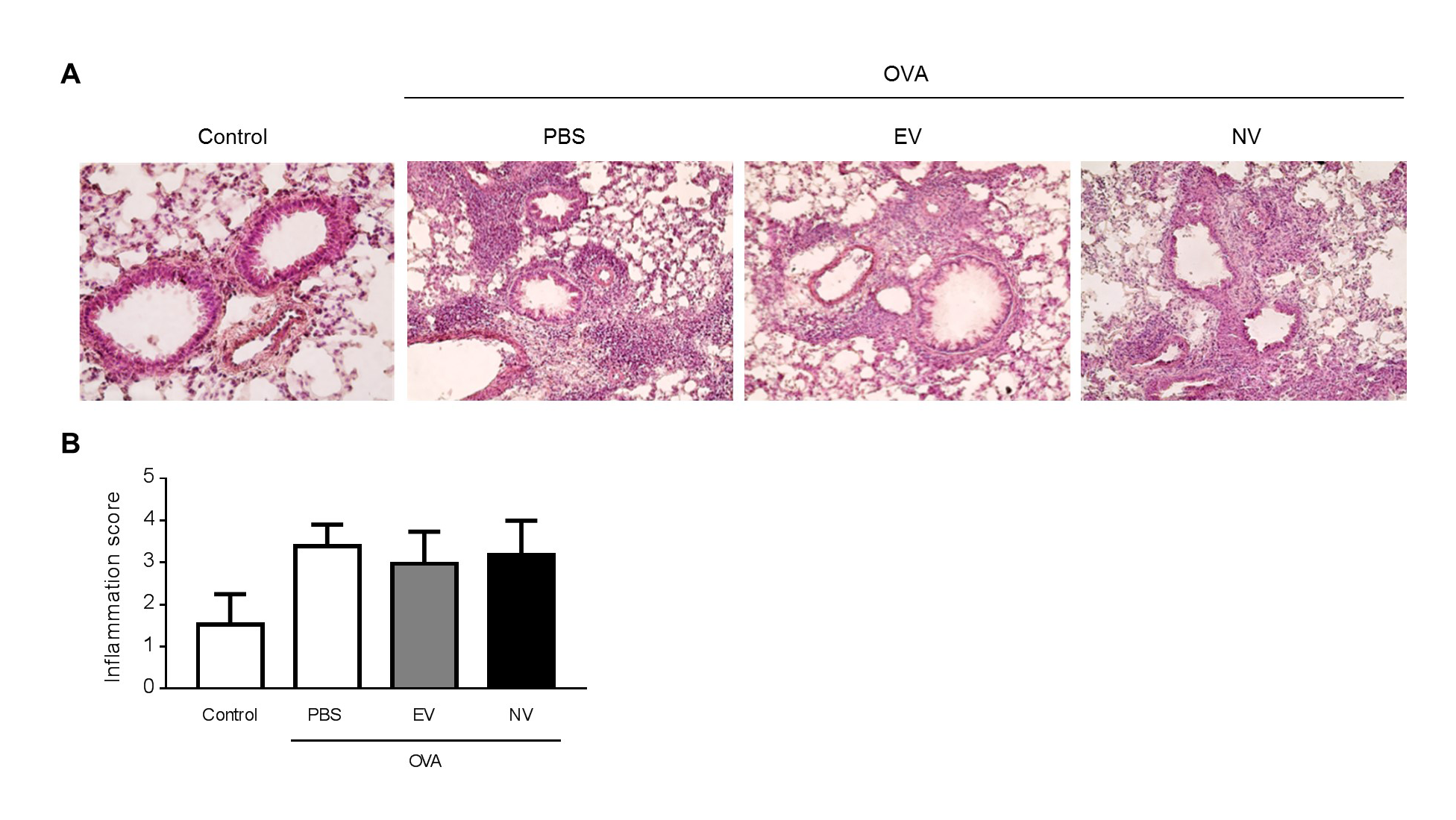
**

**Figure S3** Effects of EV and NV on airway inflammation in the asthma model. **A, B** Hematoxylin and eosin-stained lung sections (**A**) and inflammation scores (**B**) in animals challenged with OVA and subsequently treated with EV or NV. Magnifications: 400×. Data are presented as the mean ± SEM.


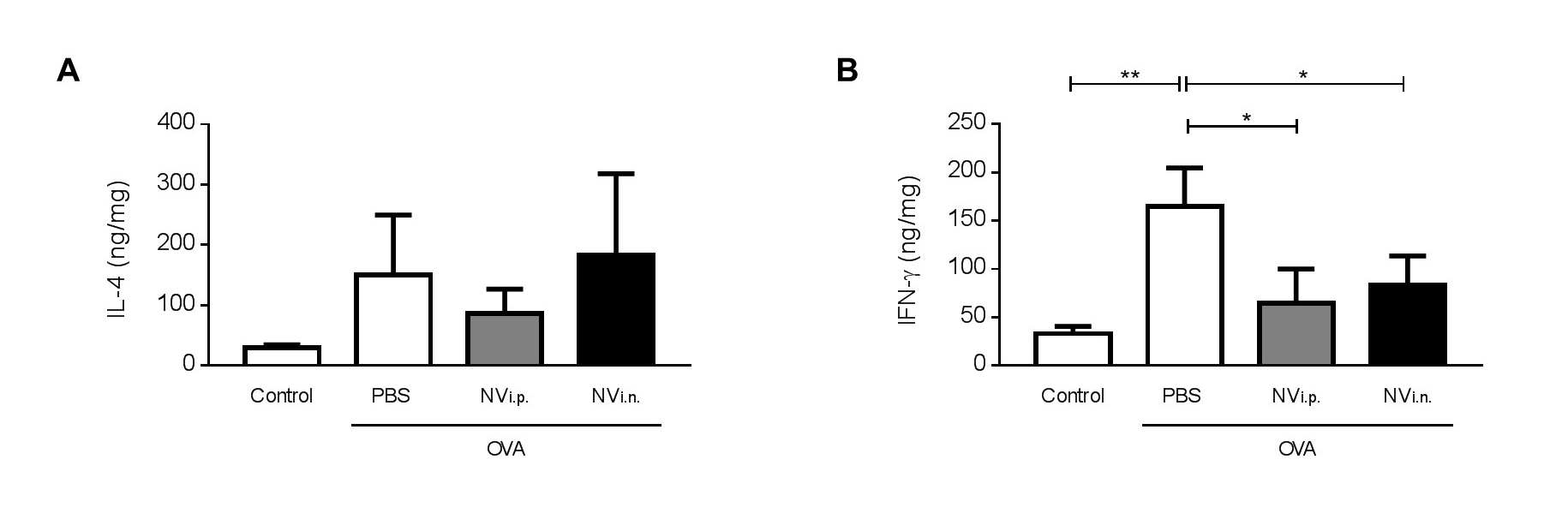


**Figure S4** The different cytokine profiles in the lungs of NV-treated mice depending on the administration route. **A, B** The levels of IL-4 (**A**) and IFN-γ (**B**) in the lung tissues. Data are presented as the mean ± SEM. **P* < 0.05, ***P* < 0.01 by one-way ANOVA with Tukey’s post test (n = 5).


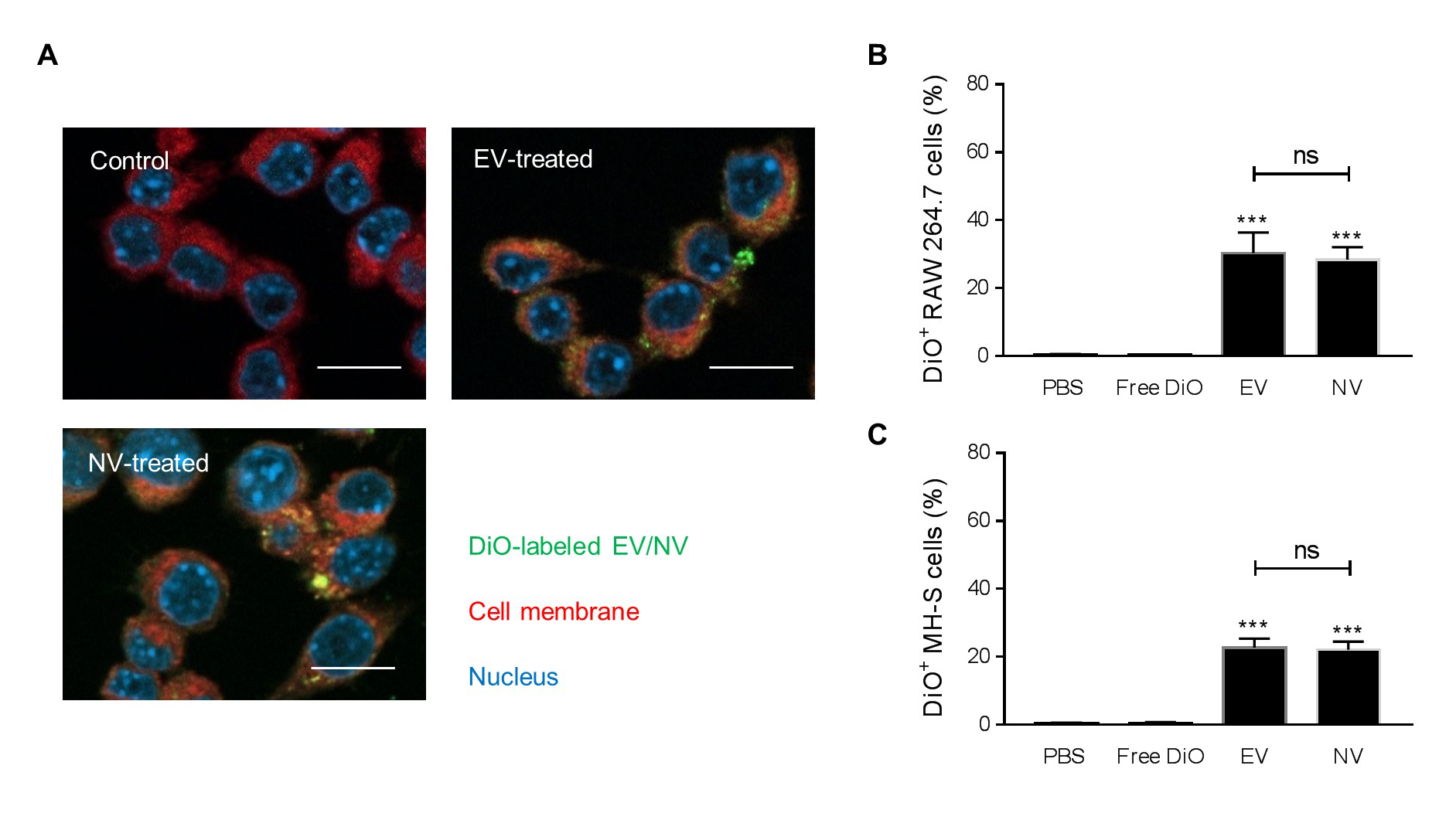


**Figure S5** The uptake profile of EV or NV in the macrophage cell line. **A** DiO-labeled EV or NV (green) was treated to RAW 264.7 cells for 6 h. Cell membranes (red) and nucleus (blue) were stained with Cellmask Deep Red and DAPI. Scale bars, 10 µm. **B, C** The uptake efficiency of both vesicles by RAW 264.7 cells (**B**) and MH-S cells (**C**) was analyzed by flow cytometry. The results are indicated by the relative percentage of DiO-positive cells. Data are presented as the mean ± SEM. ****P* < 0.001; ns, not significant, by one-way ANOVA with Tukey’s post test (n = 5).


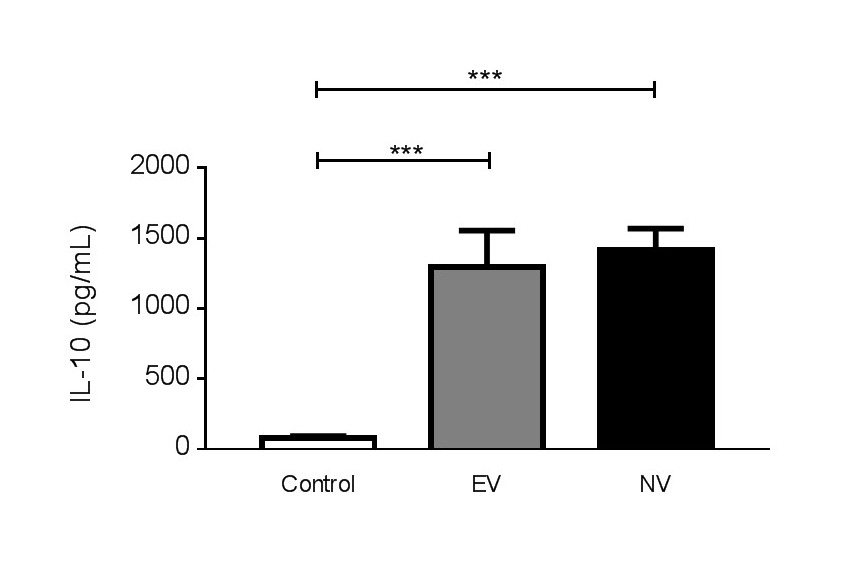


**Figure S6** IL-10 production by EV or NV in the macrophage cell line. RAW 264.7 cells were incubated with EV or NV (10^9^) for 24 h, and then the concentration of IL-10 in the conditioned medium was measured. Data are presented as the mean ± SEM. ****P* < 0.001 by one-way ANOVA with Tukey’s post test (n = 3).
